# The Annoying Nature of Snoring Sounds Is Not Only about Intensity: A Pilot Study on Exposed Test Subjects

**DOI:** 10.3390/jcm12072630

**Published:** 2023-03-31

**Authors:** Zhengfei Huang, Ghizlane Aarab, Madeline J. L. Ravesloot, Nico de Vries, Antonius A. J. Hilgevoord, Frank Lobbezoo

**Affiliations:** 1 Department of Orofacial Pain and Dysfunction, Academic Center for Dentistry Amsterdam (ACTA), University of Amsterdam and Vrije Universiteit Amsterdam, 1081 HV Amsterdam, The Netherlands; 2 Department of Clinical Neurophysiology, OLVG, 1061 AE Amsterdam, The Netherlands; 3 Department of Otorhinolaryngology—Head and Neck Surgery, OLVG, 1061 AE Amsterdam, The Netherlands; 4 Department of Otorhinolaryngology—Head and Neck Surgery, Antwerp University Hospital (UZA), 2650 Antwerp, Belgium

**Keywords:** snoring sound, acoustic characteristics, annoyance, heart rate, listener

## Abstract

This study aims to assess (i) which acoustic characteristics of snoring sounds are associated with the annoying nature of snoring sounds; (ii) whether listeners’ heart rates correlate with their perceived annoyance; and (iii) whether perceived annoyance is different between listeners with different experiences with their bedpartners’ snoring sounds. Six snoring epochs with distinct acoustic characteristics (viz., reference, high pitch, high intensity, short interval, irregular intensity, and irregular intervals) were collected from snoring patients. Twenty physicians and technicians were involved in the healthcare of snoring patients, and were divided into three groups based on personal experience with their bedpartners’ snoring sounds (viz., non-snoring, snoring but not annoying, and snoring and annoying). The test subjects listened to each epoch and rated its level of annoyance. Listeners’ heart rates were also recorded during the test using a finger plethysmograph. Within the limitations of this study, it was found that, compared with other snoring sounds, snoring sounds with high intensity and irregularity were associated with higher perceived annoyance. However, higher perceived annoyance of snoring sound was not reflected in heart rate-related parameters. In addition, listeners’ personal experiences do not seem to affect their perceived annoyance.

## 1. Introduction

Snoring is characterized by audible vibrations of the upper airway during respiration in sleep [1]. It is one of the most common sleep disorders, affecting up to 78% of adult men and 59% of adult women [2]. Because snoring sounds mainly impair the sleep quality of snorers’ bedpartners rather than snorers’ sleep quality [3], complaints from bedpartners are the main reason for snorers to seek treatment [4]. Previous studies even found that snoring is associated with marital disharmony [5,6].

The annoyance perceived by snorers’ bedpartners is mainly determined by two aspects. The first is difficulty falling asleep and maintaining sleep due to the snoring sound [7]. Second, snoring sounds can trigger a direct, unconscious autonomic stress response [8] associated with the release of stress hormones (e.g., catecholamines) [9]. These stress hormones may lead to autonomic changes like an increase in heart rate [8,9]. This stress response may additionally account for the reported annoyance of snorers’ bedpartners.

Objective indices, such as the snoring index (i.e., the number of snoring events per hour during sleep) and snoring sound intensity (i.e., sound pressure level; decibel [dB]), are generally used both clinically and for research to describe snoring. Given the importance of the subjective annoyance level, as perceived by bedpartners, researchers also explored the possibility to subjectively evaluate snoring [10,11]. Previous studies investigated the association between the acoustic characteristics of snoring sound and subjective annoyance level, and reported conflicting results [12,13,14,15,16]. Fischer et al. [12] found that a score calculated based on the loudness and roughness of snoring sound showed a highly significant correlation with perceived annoyance. Another study from Fischer et al. [13] reported that mean sound energy over the entire night showed a significant association with bedpartners’ assessments of improvement in snoring severity after treatment. In addition, Rohrmeier et al. [16] found that perceived annoyance showed highly significant corrections with the mean A-weighted sound pressure level (dBA) and the 5th percentile of loudness. Regarding the weak and moderate associations, Hoffstein et al. [15] concluded that snoring-related annoyance is in the ear of the beholder. Importantly, Dreher et al. [14] concluded that a listener’s noise sensitivity and sleep quality are at least equally relevant for the snoring-related annoyance as the snoring sound itself, suggesting that listeners with different experiences with their bedpartners’ snoring sounds, as an indicator of different noise sensitivities and sleep qualities, may have different perceived annoyance of the same snoring sound. However, no previous study has investigated whether perceived annoyance is different between listeners with different experiences with their bedpartners’ snoring sounds. In addition, previous studies mainly focused on the intensity of snoring sound, but available evidence suggests that other acoustic characteristics of snoring sound, such as pitch (hertz; Hz), may also be related to the annoying nature [7,15].

The first aim of the present study, therefore, was to assess which acoustic characteristics of snoring sounds are associated with their annoying nature by investigating the difference between listeners’ perceived annoyance caused by unfamiliar snoring epochs (i.e., snoring epochs that are not from listeners’ bedpartners) with different acoustic characteristics. We hypothesized that, in addition to high intensity, other acoustic characteristics (e.g., high pitch [Hz], short intervals between snoring events, and irregularity) of snoring sounds were also associated with higher perceived annoyance level. The second aim of this study was to assess whether listeners’ heart rates correlate with their perceived annoyance, i.e., whether listeners’ heart rates during annoying snoring epochs are higher than those during less annoying snoring epochs. We hypothesized that listeners’ perceived annoyance can be reflected in their heart rates. The third aim was to assess whether perceived annoyance is different between listeners with non-snoring bedpartners (NonSB), listeners with snoring bedpartners who do not find their bedpartners’ snoring sounds annoying (NASB), and listeners with snoring bedpartners who find their bedpartners’ snoring sounds annoying (ASB). We hypothesized that the acoustic characteristics of snoring sound are decisive for whether a snoring sound is annoying and listeners’ personal experiences do not play a decisive role in perceived annoyance of unfamiliar snoring sounds. As a consequence, we hypothesized that there is no difference in perceived annoyance between the three listener groups.

## 2. Materials and Methods

This observational study was approved by the OLVG (Amsterdam, The Netherlands) ethical committee (study number: WO 19.079). Patients who underwent an overnight polysomnography (PSG) at the Department of Neurology/Clinical Neurophysiology of OLVG West for snoring and potential OSA between July 2020 and December 2021 were included in this study. All patients were asked for permission to use their data for scientific research. Only data from patients who provided informed consent were included for further study.

### 2.1. Snoring Sound Acquisition

Polysomnograms were performed according to the standard protocol of the hospital, including electrooculogram (EOG), electroencephalogram (EEG), electrocardiogram (ECG), electromyogram (EMG), oronasal airflow, thoraco-abdominal movement, oxygen saturation (SPO2), pulse, and position monitoring. For the overnight sound recording, a digital audio recorder with a non-contact microphone (ZOOM H5, Zoom Corporation, Tokyo, Japan) was used to record snoring sounds during PSG. The recorder was placed on a microphone stand (one meter high) at the snorer’s side and head end of the bed, with the base of the microphone stand approximately 20 cm from the bed to prevent direct contact noises. The microphone was pointed at the snorer. Before every recording, the audio recorder was calibrated using a sound pressure calibrator (Testo 0554.0452, Testo Ltd., Alton Hampshire, UK), which produced a reference sound pressure level (94 dB [0 dB = 20 µPa], 1 kHz sine wave). After recording, the original recordings with a sampling frequency of 44.1 kHz were digitally down-sampled to 22 kHz in Audacity (2.3.2, Audacity^®^, Renton, WA, USA), an audio software, as a compromise between the desired frequency range and data size and the processing time.

### 2.2. Selection of Snoring Epochs with Different Acoustic Characteristics

**Figure 1:**
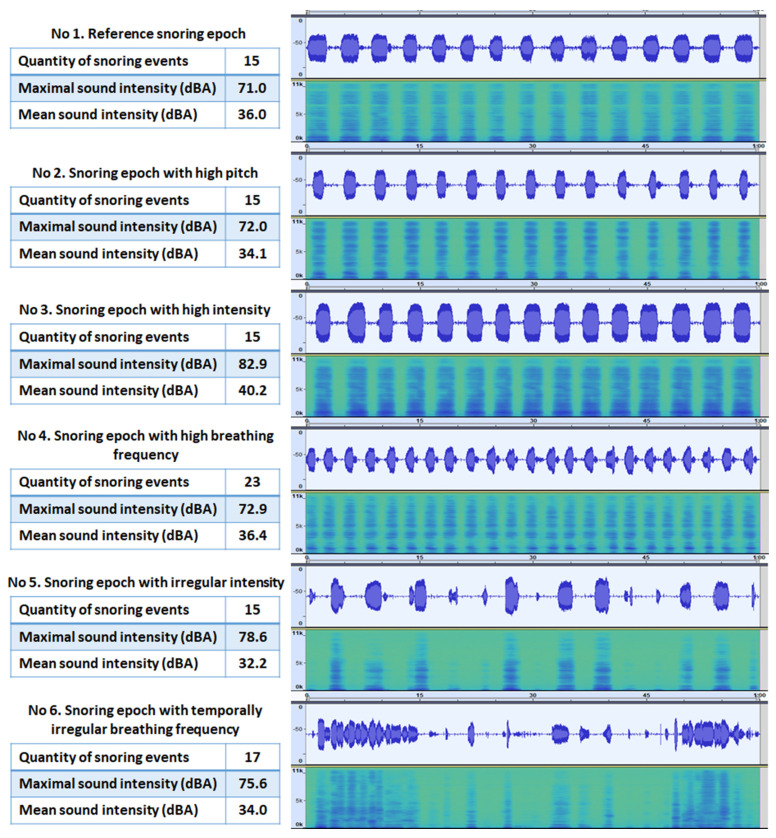
The six snoring epochs used in this study. Descriptive information of the snoring epochs (viz., quantity of snoring events, maximal sound intensity, and mean sound intensity) is shown on the left side. On the right side, the waveforms of the snoring epochs are shown in the upper panels (with white background); the amplitudes of waveforms represent the intensities of snoring sounds. In the lower panels (with green background), the spectra show the frequency distribution (0–11 kHz) of the snoring epochs. The blue horizontal lines represent the power of the sound, i.e., the bluer the horizontal lines at certain frequency, the higher power the sound has at this frequency.

In this study, snoring epochs (viz., snoring sound clips) consisting of snoring events with different acoustic characteristics were used as samples for scoring. The selection of snoring epochs was performed in R (Vienna University of Economics and Business, Austria), a programming language, and the visualization of sound profiles and spectrograms of snoring epochs was achieved in Audacity. To avoid the potential influence of short-term auditory fatigue (i.e., a temporary rise in the auditory threshold [17]) on listeners’ perception of snoring sounds, and to show the variation of snoring sound over time, a duration of 60 s was arbitrarily considered the best compromise for the snoring epochs. Snoring sound is generally analyzed in three domains, viz., the temporal domain, the intensity domain, and the frequency domain. From the sound recordings of the included patients, six snoring epochs that represent snoring sounds observed in the included patients were extracted. The six snoring epochs covered distinct characteristics in the above-mentioned three domains (Figure 1; Supplementary Materials Recordings S1–S6):

1. Snoring epoch consisting of snoring events with relatively medium (40–70 dBA; based on the population and recording setting in the present study) intensity and the most common frequency distribution (high power in relatively low frequency components; reference snoring epoch);
2. Snoring epoch consisting of snoring events with high pitch (i.e., high power in relatively high frequency components; Hz);
3. Snoring epoch consisting of snoring events with high intensity (>70 dBA);
4. Snoring epoch with high breathing frequency (i.e., more snoring events and shorter time intervals between snoring events);
5. Snoring epoch consisting of snoring events with irregular intensity;
6. Snoring epoch with temporally irregular breathing frequency (i.e., the durations of the snoring events and of the time intervals between events are irregular).

The selected six snoring epochs were extracted and assembled in one continuous audio file for the test. To avoid the potential temporary threshold shift and to give listeners enough time to score each snoring epoch, a 30 s silent epoch was inserted following each snoring epoch. In addition, it is possible that listeners find the last snoring epoch in each audio file the most annoying since listeners gradually get tired of the snoring sound. To avoid this potential bias, the six snoring epochs were randomly assembled in five audio files with different orders.

### 2.3. Listeners Recruitment

**Figure 2:**
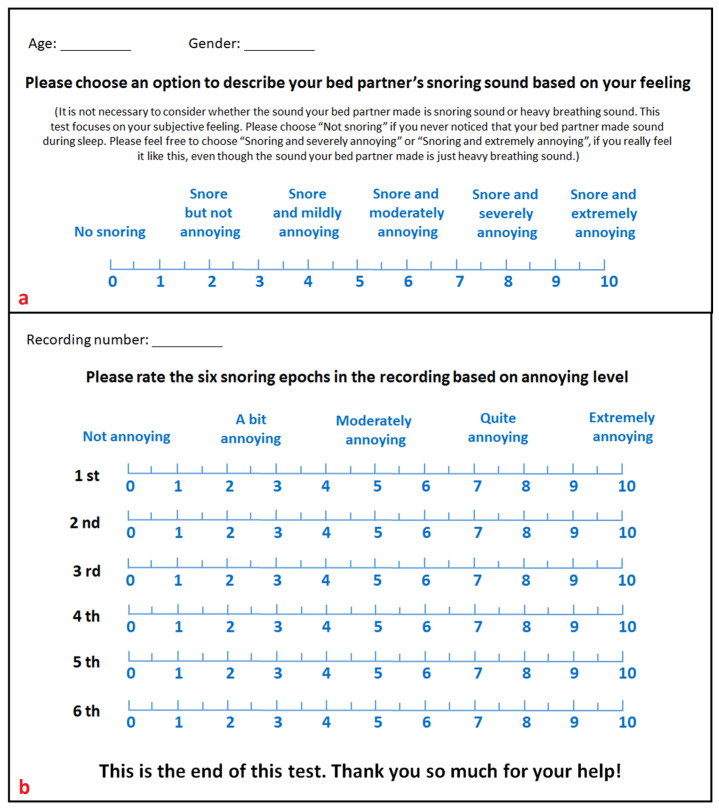
Questionnaire for this study. This questionnaire consisted of two parts, viz., (**a**,**b**). Part (**a**) was used before the test. Listeners were asked to rate their own bedpartners’ snoring sounds and were divided into different groups based on their answers; Part (**b**) was used for the test. Recording number indicated which of the five audio files with randomly sorted snoring epochs was used for the test. In addition, listeners were asked to score the six snoring epochs: 0 meant not annoying and 10 meant extremely annoying.

Listeners were recruited amongst clinicians and technicians at the Department of Otorhinolaryngology and the Department of Neurology/Clinical Neurophysiology (OLVG, Amsterdam, The Netherlands). All clinicians and technicians who were involved in the healthcare of snoring patients were approached for this study. Clinicians and technicians who were willing to participate in this study and had normal hearing were included as listeners. It needs to be noted that their expertise in snoring and experience with snoring sounds may affect their perception of snoring sounds. All listeners were unaware of the identity of the participants from whom the six snoring epochs were selected. After recruitment, listeners were asked to rate their own bedpartners’ snoring sounds using a VAS (0–10; Figure 2a) and were divided into three groups based on their answers. Listeners who selected “Not snoring” were categorized as NonSB; listeners who selected “Snoring but not annoying” were categorized as NASB; and listeners who selected “Snoring and mildly annoying”, “Snoring and moderately annoying”, “Snoring and severely annoying”, or “Snoring and extremely annoying” were categorized as ASB.

### 2.4. Evaluation of Snoring Epochs

To avoid the difference in perceived snoring sound intensity caused by different distances between the audio player and listeners, a small office was used for the test and only one listener was tested at a time. Sound intensity was calibrated based on the known correspondence between the maximal sound intensity and the amplitude of the sound wave. In addition, to ensure that all listeners were on a comparable level of alertness, all tests were performed between 12:30 and 14:30. Prior to the test, listeners were instructed to close their eyes and imagine that they were lying in bed, attempting to fall asleep. During the test, a portable heart rate monitor (PO 80, Beurer GmbH, Uttenweiler, Germany; reporting frequency of heart rate = 1 Hz) with a data export function was used to record the heart rate of the listener from the finger plethysmograph. For scoring of the snoring epochs (0–10; Figure 2b), listeners were instructed to give a score to rate their level of annoyance after listening to each individual snoring epoch.

After the test, data from the questionnaires were extracted and the listener’s heart rate recording was exported for analysis. The mean heart rate during each 60 s snoring epoch was calculated. In addition, skewness of the distribution of heart rates during each 60 s snoring epoch was tested. Furthermore, heart rate variability during a 60 s snoring epoch was evaluated using the root mean square of successive differences (RMSSD) of heart rate [18].

### 2.5. Statistics

Concerning the descriptive statistics, nominal data were presented as percentages. Given the relatively small sample size in this pilot study, continuous data were presented as the median and interquartile range (IQR), and non-parametric tests were used. For the first aim, given that the measurements were repeated (i.e., every participant scored six snoring epochs), there might be a correlation between the measurements. Therefore, the Friedman test was used to assess the difference in perceived annoyance between snoring epochs. When a significant difference (*p* < 0.05) was observed, the Wilcoxon signed-rank test was used to evaluate the difference between every two groups. For the second aim, the Kruskal–Wallis test was used to assess the difference in heart rate-related parameters (viz., mean heart rate, skewness, and RMSSD) during snoring epochs. When a significant difference (*p* < 0.05) was observed, the Mann–Whitney U test was used to evaluate the difference between every two groups. The same procedure was performed for the third aim, which was to assess the difference in perceived annoyance between the three listener groups (viz., NonSB, NASB, and ASB). Bonferroni adjustment for multiple comparisons was used for the second and third aims [19]. All analyses were conducted using the IBM SPSS Statistics 27 software package (IBM Corp., Chicago, IL, USA).

## 3. Results

A total of 20 clinicians and technicians were included as listeners in this study. Of the 20 listeners, eight were categorized as NonSB, five were NASB, and seven were ASB. The descriptive statistics of listeners’ demographic parameters, perceived annoyance, and heart rate-related parameters are shown in Table 1. Notably, a significant difference in age between NonSB and NASB was observed (*p* = 0.01). There was no significant difference between the three listener groups in gender (*p* = 0.26).

For the first aim, a significant difference (*p* < 0.01) was observed in perceived annoyance between the snoring epochs. The results of the Wilcoxon signed-rank tests indicated that the perceived annoyance caused by the snoring epoch with high intensity was significantly higher than that caused by other snoring epochs (*p* < 0.05). In addition, the perceived annoyance caused by the snoring epoch with irregular intensity and the snoring epoch with temporally irregular breathing frequency was significantly higher than that caused by the reference snoring epoch, the snoring epoch with high pitch, and the snoring epoch with high breathing frequency (*p* < 0.05).

For the second aim, no significant difference in heart rate-related parameters (viz., mean heart rate [*p* = 0.3], skewness [*p* = 0.4], and RMSSD [*p* = 0.1]) between snoring epochs was observed, suggesting that the difference in listeners’ perceived annoyance cannot be reflected in their heart rates.

For the third aim (Table 2), despite the significant difference in age, no significant difference in perceived annoyance could be demonstrated between the three listener groups (NonSB, NASB, and ASB; *p* > 0.05). Analyses of the heart rate-related parameters confirmed this result (*p* > 0.05).

## 4. Discussion

This study aimed to assess (i) which acoustic characteristics of snoring sounds are associated with the annoying nature of snoring sounds; (ii) whether listeners’ heart rates correlate with their perceived annoyance; and (iii) whether perceived annoyance is different between listeners with different experiences with their bedpartners’ snoring sounds. It was found that, in addition to the intensity of snoring sound, the irregularity of the intensity of snoring sound and the irregularity of intervals between snoring sounds were also associated with listeners’ perceived annoyance. However, no significant difference in heart rate-related parameters was observed between snoring epochs. In addition, no significant differences in perceived annoyance and heart rate-related parameters were observed between the listener groups.

As the main finding, irregularity of snoring sound was found to be associated with listeners’ perceived annoyance. During the test, almost all listeners indicated that the unpredictable nature of snoring epochs with irregular intensity and interval would make them anxious and stop them from falling asleep, because they did not know what would happen next. Although the snoring epoch with high intensity was found to be more annoying than others, several listeners believed that, if they were exposed to such noise for months or years, they might get used to this kind of regular noise and might automatically filter it out in the same way that they filter out the noise of traffic, for example. As for other acoustic characteristics, the difference between the reference snoring epoch and the snoring epoch with a high pitch was clearly noticeable, but a higher pitch does not seem to be associated with higher perceived annoyance. For the snoring epoch with high breathing frequency, shorter intervals between snoring sounds were expected to be associated with higher perceived annoyance as this might cause more anxiety in listeners, but such a result was not observed. This may be because the regularity of this snoring epoch makes it less annoying than expected, suggesting that the regularity of snoring sound is more perceivable than its repetition rate. Based on these findings, it is suggested that when evaluating the severity of snoring and the efficacy of snoring-related treatment, in addition to the intensity of snoring sounds, clinicians should also take the irregularity of snoring sounds into consideration. In clinical practice, we see that snoring is most prevalent during sleep stage III and rapid eye movement (REM). However, during REM sleep, snoring sounds are more irregular. This suggests the importance of time series analysis of the irregularity of snoring sound in further studies on the annoying nature of snoring sound. In addition, it is not advisable to use a single quantitative parameter of snoring characteristics as an indicator of snoring severity/snoring-related treatment efficacy, or for comparison purposes.

Notably, it was considered that listeners may find the last snoring epoch in each audio file most annoying, because listeners gradually get tired of the snoring sound. We tested whether there is a cumulative effect of perceived annoyance, but an insignificant result was observed (*p* = 0.3; Supplementary Table S1).

For the second aim, although all previous studies on heart rate and noise exposure reported a positive association between heart rate and noise [8,9,20], no difference in heart rate-related parameters was observed between snoring epochs in this study. There are three possible explanations for this result. The first possible explanation is that the difference in heart rate-related parameters can only be observed between the situations with and without noise, while the difference in perceived annoyance between snoring epochs was too subtle to be reflected in heart rate-related parameters. The second possible explanation is that the difference in perceived annoyance between snoring epochs can be reflected in heart rate-related parameters, but one minute (i.e., the duration of each snoring epoch) is too short to show the difference in heart rate-related parameters. The third possible explanation is that the sample size in this study is too small to show the difference. The fourth possible explanation is that the perceived annoyance of snoring sounds was underestimated because this study was conducted during the day. When snorers’ bedpartners are trying to fall asleep at night, the same snoring sounds may cause higher perceived annoyance and stress response than during the day and, consequently, cause significant variation in heart rate. It needs to be noted that the insignificant difference in heart rate-related parameters between snoring epochs does not negate the possibility to use snorers’ bedpartners’ heart rate-related parameters as objective indices to evaluate the severity/annoying degree of snoring sounds. However, before that, further studies are needed to investigate whether there is a difference in heart rate-related parameters between the situations with and without snoring sounds, and whether the difference in perceived annoyance between snoring epochs can be reflected in heart rate-related parameters over a longer period of time.

For the third aim, as expected, for all snoring epochs, despite the significant difference in age, no significant difference in perceived annoyance was observed between the three listener groups. This finding suggested that the acoustic characteristics of a snoring sound are decisive for whether the snoring sound is annoying, and listeners’ personal experiences do not play a decisive role in perceived annoyance of unfamiliar snoring sounds. In addition, in line with a previous study [21], the present study suggested that the perceived annoyance of snorers’ bedpartners may be used as a reliable assessment of the severity/annoying degree of snoring. However, some other studies suggested the potential influence of listeners’ subjectivities on perceived annoyance of snoring sound and concluded that subjectively evaluated snoring severity should be interpreted with caution [13,14]. Regarding the conflicting results on listeners’ subjectivities and perceived annoyance of snoring sound, this may be due to different study designs. For example, for each included snorer, Dreher et al. extracted a typical snoring event and repeated this typical snoring event three times to assemble a snoring epoch for audible evaluation [14]. Besides the difficulty in evaluating the annoying nature of snoring sounds in such a short period of time, repeating a typical snoring event three times as the snoring epoch for audible evaluation would lose the important characteristics of authentic snoring sounds, such as the irregularity of snoring sounds, which was found to be associated with higher perceived annoyance in the present study. Further well-designed studies with more listeners are needed to investigate the effect of listeners’ subjectivities on perceived annoyance of snoring sound.

This study has several limitations. First, as a pilot study, this study only included 20 listeners, and non-parametric tests had to be employed for analyses, which compromised the statistical power of the results. In addition, the difference in age between the three listener groups might also have influenced the result of the third aim to some extent. Second, only physicians and technicians who were involved in the healthcare of snoring patients were included as listeners in the present study. As mentioned above, the expertise in snoring and the experience with snoring sounds might have had unknown effects on the results. The authors, therefore, encourage other researchers to repeat this study with people without a professional background in snoring. Last, the present study was conducted during the day and used six short snoring epochs to investigate which acoustic characteristics of snoring sound are associated with the annoying nature of snoring sound. Further studies are recommended to be conducted in a real-life scenario, where listeners would hear snoring sounds from their bedpartners at night in the bedroom, to test the findings from this laboratory-based pilot study. In addition, it is recommended to use longer snoring epochs to investigate the association between listeners’ heart rates and their perceived annoyance.

## 5. Conclusions

Within the limitations of this study, compared with other snoring sounds, snoring sounds with high intensity and irregularity were associated with higher perceived annoyance. However, higher perceived annoyance of snoring sound was not reflected in heart rate-related parameters. Finally, listeners’ personal experiences do not seem to affect their perceived annoyance.

## Figures and Tables

**Table 1:** The descriptive statistics of listeners’ demographic parameters, perceived annoyance, and heart rate-related parameters.

	Total (*n* = 20)	NonSB (*n* = 8)	NASB (*n* = 5)	ASB (*n* = 7)
Demographic parameters				
Age (years; median [IQR])	42.0 (30.5–57.5)	32.5 (27.0–39.5)	49.0 (46.5–64.0)	43.0 (29.0–60.0)
Gender (*n*; %)				
- Male	7 (35.0%)	3 (37.5%)	3 (60.0%)	1 (14.3%)
- Female	13 (65.0%)	5 (62.5%)	2 (40.0%)	6 (85.7%)
Perceived annoyance and heart rate-related parameters by snoring epochs				
Reference snoring epoch				
- Perceived annoyance (median [IQR])	7.3 (6.0–8.0)	7.5 (4.0–8.0)	8.0 (6.0–8.0)	7.0 (6.0–8.0)
- Mean heart rate (bpm; median [IQR])	67.0 (61.5–75.5)	64.7 (53.0–70.9)	62.2 (54.5–84.4)	70.5 (66.2–76.6)
- Skewness (median [IQR])	0.3 (−0.1–0.7)	0.3 (0.0–0.64)	0.0 (−0.4–1.2)	0.5 (−0.1–0.9)
- RMSSD (median [IQR])	5.7 (5.0–7.4)	5.9 (5.0–6.5)	5.4 (4.4–8.0)	5.6 (5.2–7.5)
Snoring epoch with high pitch				
- Perceived annoyance (median [IQR])	7.0 (6.0–8.0)	7.3 (6.3–8.4)	6.5 (4.3–8.3)	7.0 (6.0–8.0)
- Mean heart rate (bpm; median [IQR])	68.0 (60.0–77.7)	66.1 (54.2–72.0)	62.4 (54.8–84.4)	69.7 (67.6–81.1)
- Skewness (median [IQR])	0.2 (0.1–0.6)	0.2 (0.0–0.5)	0.5 (−0.1–0.7)	0.2 (0.2–1.0)
- RMSSD (median [IQR])	6.2 (5.5–7.4)	6.5 (6.0–7.7)	5.7 (4.7–8.4)	5.9 (5.5–7.2)
Snoring epoch with high intensity				
- Perceived annoyance (median [IQR])	9.3 (8.0–10.0)	9.3 (8.0–10.0)	8.5 (6.3–9.5)	10.0 (9.0–10.0)
- Mean heart rate (bpm; median [IQR])	67.7 (61.7–77.8)	63.5 (54.5–71.7)	62.7 (54.3–86.9)	75.0 (68.2–82.1)
- Skewness (median [IQR])	0.2 (−0.1–0.7)	0.1 (−0.1–0.3)	0.8 (−0.2–0.9)	0.3 (−0.5–0.5)
- RMSSD (median [IQR])	6.1 (4.9–8.2)	6.9 (4.4–8.1)	6.2 (5.2–9.3)	5.5 (4.8–10.1)
Snoring epoch with high breathing frequency				
- Perceived annoyance (median [IQR])	7.0 (5.0–8.0)	6.0 (4.3–7.8)	7.0 (4.8–8.5)	7.0 (5.0–8.0)
- Mean heart rate (bpm; median [IQR])	66.5 (60.9–78.1)	65.6 (55.0–72.9)	62.5 (55.7–84.6)	67.8 (64.9–83.4)
- Skewness (median [IQR])	0.4 (−0.1–0.8)	0.4 (−0.2–0.8)	−0.1 (−0.5–1.1)	0.3 (0.0–0.9)
- RMSSD (median [IQR])	5.5 (4.5–7.2)	5.2 (4.2–7.2)	6.1 (5.1–9.7)	5.3 (4.5–5.9)
Snoring epoch with irregular intensity				
- Perceived annoyance (median [IQR])	8.0 (7.1–9.0)	8.0 (7.3–9.0)	7.5 (6.8–8.0)	9.0 (8.0–9.0)
- Mean heart rate (bpm; median [IQR])	66.6 (61.2–77.3)	65.3 (54.1–70.9)	64.7 (54.2–85.0)	69.3 (62.9–82.1)
- Skewness (median [IQR])	0.2 (−0.1–0.6)	0.3 (−0.3–0.7)	0.2 (−0.1–0.4)	0.0 (−0.1–0.9)
- RMSSD (median [IQR])	5.8 (4.9–7.1)	5.8 (5.0–6.4)	5.4 (3.2–7.2)	6.1 (5.0–7.9)
Snoring epoch with temporally irregular breathing frequency				
- Perceived annoyance (median [IQR])	8.5 (7.1–9.0)	8.5 (7.1–9.8)	7.5 (5.5–9.3)	9.0 (8.0–9.0)
- Mean heart rate (bpm; median [IQR])	68.7 (61.0–76.6)	66.6 (51.2–72.4)	61.6 (54.9–85.5)	73.8 (65.7–82.4)
- Skewness (median [IQR])	0.4 (0.0–0.6)	0.3 (0.0–0.7)	0.2 (−0.3–1.1)	0.6 (−0.3–0.6)
- RMSSD (median [IQR])	6.3 (5.4–7.0)	6.3 (5.7–7.3)	6.1 (4.9–10.7)	6.3 (4.8–6.9)

Note. ASB = listeners with snoring bedpartners and find their bedpartners’ snoring sounds annoying; bpm = beats per minute; IQR = interquartile range; NASB = listeners with snoring bedpartners but do not find their bedpartners’ snoring sounds annoying; NonSB = listeners with non-snoring bedpartners; RMSSD = root mean square of successive differences.

**Table 2:** The difference in perceived annoyance and heart rate-related parameters between three listener groups.

Snoring Epoch	Parameter	*p* Value (NonSB vs. NASB vs. ASB)
Reference snoring epoch	Perceived annoyance	0.3
	Mean heart rate	0.6
	Skewness	0.7
	RMSSD	0.8
Snoring epoch with high pitch	Perceived annoyance	0.5
	Mean heart rate	0.6
	Skewness	0.8
	RMSSD	0.6
Snoring epoch with high intensity	Perceived annoyance	0.5
	Mean heart rate	0.4
	Skewness	0.5
	RMSSD	0.8
Snoring epoch with high breathing frequency	Perceived annoyance	0.5
	Mean heart rate	0.6
	Skewness	0.6
	RMSSD	0.2
Snoring epoch with irregular intensity	Perceived annoyance	0.4
	Mean heart rate	0.7
	Skewness	0.9
	RMSSD	0.8
Snoring epoch with temporally irregular breathing frequency	Perceived annoyance	0.9
	Mean heart rate	0.4
	Skewness	0.8
	RMSSD	0.8

Note. ASB = listeners with snoring bedpartners and find their bedpartners’ snoring sounds annoying; NASB = listeners with snoring bedpartners but do not find their bedpartners’ snoring sounds annoying; NonSB = listeners with non-snoring bedpartners; RMSSD = root mean square of successive differences.

## Data Availability

The data presented in this study are available on request from the corresponding author.

## Supplementary Materials

The following supporting information can be downloaded at: https://www.mdpi.com/article/10.3390/jcm12072630/s1, Table S1: The correlation between the order of snoring epoch and listeners’ perceived annoyance; Snoring Sound Recordings S1–S6.
