# Supplementary material for: The Annoying Nature of Snoring Sounds Is Not Only about Intensity: A Pilot Study on Exposed Test Subjects
Source: J Clin Med. 2023 Mar 31;12(7):2630. doi: 10.3390/jcm12072630 (PMC10095263; doi:10.3390/jcm12072630)
Supplement: Supplementary file 1 [file jcm-12-02630-s001.zip › Table S1.pdf]

Supplementary material

Table S1. The correlation between the order of snoring epoch and listeners' perceived annoyance

Correlations

			score	order
Spearman's rho	score	Correlation Coefficient	1.000	0.486
		Sig. （2-tailed）		0.329
		N	6	6
	order	Correlation Coefficient	0.486	1.000
		Sig. （2-tailed）	0.329	
		N	6	6
